# Exposure to N,N-diethyl-*m*-toluamide and cardiovascular diseases in adults

**DOI:** 10.3389/fpubh.2022.922005

**Published:** 2022-10-03

**Authors:** Shiwei Yan, Jianing Wang, Jiaxu Xu, Wenbo Jiang, Menglin Xiong, Ziteng Cao, Yu Wang, Ziqi Wang, Tongfang Zhang, Zheng Wang, Changhao Sun, Shaoying Hou, Wei Wei

**Affiliations:** ^1^Department of Nutrition and Food Hygiene, College of Public Health, Harbin Medical University, Harbin, China; ^2^Department of Cerebrovascular Disease, The Fifth Affiliated Hospital, Sun Yat-Sen University, Zhuhai, China

**Keywords:** DEET, CVD, CHD, DCBA, NHANES

## Abstract

Although growing evidence suggests that N,N-diethyl-*m-*toluamide (DEET) has adverse effects on public health, the relationship of DEET with cardiovascular disease (CVD) is still largely unknown. The purpose of this study was, therefore, to evaluate the association between DEET exposure and total and specific CVD among the US adults. In this cross-sectional study, a total of 5,972 participants were selected from the National Health and Nutrition Examination Survey (NHANES) 2007–2014. CVD was defined as a combination of congestive heart failure (CHF), coronary heart disease (CHD), angina, heart attack, or stroke. Logistic regression models were used to evaluate the association between DEET metabolites and the risks of total and specific CVD. Compared to the lowest quartile, 3-(diethylcarbamoyl) benzoic acid (DCBA) in the highest quartile was associated with the increased risks of CVD (odds ratio [OR]: 1.32, 95% CI: 1.03–1.68, *P* for trend = 0.025) and CHD (OR: 1.57, 95% CI: 1.10–2.25, *P* for trend = 0.017), after adjustment for potential covariates. Nevertheless, exposure to DCBA was not significantly associated with heart attack, CHF, angina, and stroke. Further studies are required to confirm these findings and identify the underlying mechanisms.

## Introduction

N,N-diethyl-*m*-toluamide (DEET), the major active ingredient of common topical insect repellents, was first developed by the US Department of Agriculture as protection for the military in 1946 and registered for general public use in 1957 (1, 2). Numerous studies have confirmed that DEET is the most effective repellent for biting insects, mosquitoes, and ticks (3–6). DEET-containing products exist in many different forms such as liquids, sprays, roll-ons, and sticks. It is estimated that the average annual US yield is 1,800 tons of DEET and almost 30% of the population uses insect repellent products containing DEET (7–9), resulting in human exposure. Furthermore, DEET has been detected in streams, surface waters, and groundwater throughout the US, albeit at trace levels. Due to its mobility and persistence, there are concerns that DEET in aquatic environments may pose a series of public health questions.

The US Environmental Protection Agency declares that the risks of DEET to human health or the environment are clearly exaggerated, and reasonable use might effectively reduce the incidence of vector-borne diseases (VBDs) (10). Commercial products containing DEET are available in the concentrations of 4–100%; there was long-lasting protection in higher concentrations, while increasing the concentration above 50% does not improve efficacy. Using the concentration of ≥20% in adults for protection against insect bites was recommended by the US Centers for Disease Control and Prevention (CDC).

Recently, the safety profile topics surrounding DEET have attracted increasing attention and controversy. It is reported that either chronic or acute exposure to DEET can result in some adverse effects in children and adults including skin irritations and neurological and cardiovascular disorders (11–16). Nevertheless, many epidemiological experts insist that there is no problem in using DEET in daily life if applied following the labeled instructions. Furthermore, animal experiments and population studies found that low-dose DEET exposure showed either no symptoms or only minor symptoms that were rapidly resolved (14, 17).

Although the influence of DEET on human health appears to be balanced between control of VBD and increased evidence of adverse effects in humans, very few studies draw definitive conclusions about the risk assessment of DEET. Hence, we evaluated the association between DEET exposure and the risk of cardiovascular disease (CVD) using data from the National Health and Nutrition Examination Survey (NHANES).

## Materials and methods study population

The NHANES is a nationally representative health survey of the non-institutionalized United States population using a stratified, multistage probability design. Detailed information on NHANES has been previously provided (18). Briefly, adults (aged ≥ 30 years) who participated in NHANES from 2007 to 2014 were selected for this study (Figure S1). After the exclusion of participants who had missing information on urinary samples and CVD outcomes, a total of 5,972 participants were included in the study. All protocols were approved by the National Center for Health Statistics Ethics Review Board and documented consent was obtained from all participants.

### DEET metabolite measurements

The urine samples were frozen at the temperature of −20°C and shipped to the division of the Environmental Health Laboratory Sciences, National Center for Environmental Health, CDC. Urinary concentrations of DEET and its two metabolites, 3-(diethylcarbamoyl) benzoic acid (DCBA) and N,N-diethyl-3-(hydroxymethyl) benzamide (DHMB), were measured by solid-phase extraction coupled with high-performance liquid chromatography-tandem mass spectrometry. As the detectable concentrations of DEET and DHMB are considerably low, only DCBA was included in our study. The lower limit of detection (LOD) for DCBA, DHMB, and DEET was 0.929 μg/L (NHANES 2007–2008) and 0.475 μg/L (NHANES 2009–2010, 2011–2012, and 2013–2014), 0.089 μg/L (NHANES 2007–2014), and 0.083 μg/L (NHANES 2007–2014), respectively. Substituted values below the LOD with LOD divided by the square root of 2.

### Covariates

Covariates, including age, gender, ethnicity, education levels, annual household income, regular exercise, current smoker, current drinker, ever told had hypertension, dyslipidemia, and diabetes were obtained through standardized questionnaires. Body weight and height were measured by a trained health technician using standardized technique equipment. Body mass index (BMI) was calculated as weight (kg) divided by the square of the height in meters (m^2^). For all questions, responses of “refused responses” or “don't know” were coded as missing. Age and natural ln-transformed creatinine concentration were treated as continuous variables. The categories of other covariates were as follows: sex (male/female), race/ethnicity (Mexican American/non-Hispanic White/non-Hispanic Black/other race), education level (< 9th grade/9–11th grade/high school graduate/GED or equivalent/some college graduate, or AA degree/college graduate or above), annual household income (<$20,000/$20,000–$45,000/$45,000–$75,000/$75,000–$100,000/>$100,000), regular exercise (yes/no), current smoker (yes/no), current drinker (yes/no), BMI (underweight <18.5 kg/m^2^/18.5 kg/m^2^ ≤ normal weight <25 kg/m^2^/25 kg/m^2^ ≤ overweight <30 kg/m^2^/30 kg/m^2^ ≤ obesity), ever told had diabetes (yes/no/borderline), and ever told had dyslipidemia or hypertension (yes/no).

### Assessment of CVD outcomes

The total CVD was defined as a combination of self-reported physician diagnoses of specific CVD, including congestive heart failure (CHF), coronary heart disease (CHD), angina, heart attack, or stroke. For the classification of CVD, if participants responded “Yes” to any of these cardiovascular symptoms represented by the following questions, he or she was considered a patient with CVD; “Has a doctor ever told you that you have CHF, CHD, angina, heart attack, or stroke?” The CVD outcome was, therefore, converted into a dichotomous variable.

### Statistical analyses

Selected demographic characteristics were presented as mean ± standard deviation (SD) for continuous variables and percentage (%) for categorical variables. The differences in demographic characteristics between the four groups classified by urinary DCBA were, respectively, tested using general linear models and χ^2^ tests for continuous and categorical variables. Considering that urinary concentration of creatinine exhibited the skewed distribution, it was naturally ln-transformed in all analyses (19). The concentrations of urinary DCBA were grouped into quartiles, and the reference category was considered to be the lowest quartile. Logistic regression models were used to calculate the odds ratio (OR) and 95% confidence interval (CI) to assess the association between urinary DCBA and the risk of CVD outcomes. We also fitted three-knot restricted cubic splines (RCS) to explore the shape of the dose–response relationship between urinary concentrations of DCBA and CVD risk. There were three models in the present analysis. Model 1 was adjusted for age, sex, and ln-transformed creatinine. Model 2 was adjusted for covariates in model 1 plus race/ethnicity, education level, annual household income, exercise regularly, current smoker, current drinker, and BMI. Model 3 was adjusted for covariates in model 2 plus diabetes, dyslipidemia, and hypertension.

Sensitivity analyses were performed to assess the robustness of our results. First, the family history of CVD (heart attack) and diabetes was additionally adjusted. Second, we performed weighted analyses to confirm whether the results were influenced by statistical weighting. In addition, stratified analyses were conducted by age (≤60 or >60 years), gender (male or female), race (non-Hispanic White or other race), income (low or high), and BMI (<30 or ≥ 30 kg/m^2^).

All statistical analyses were performed with the use of R version 3.6.2. A *P*-value of < 0.05 was considered statistically significant. The Bonferroni correction threshold was considered to account for multiple comparisons and define statistical significance (0.05/5 = 0.01 for the stratified interaction tests).

## Results

### Basic characteristics of subjects

Our analytic sample consisted of 2,876 (48.2%) men and 3,096 (51.8%) women and had a mean age of 54.2 (14.9) years. The overall prevalence of total CVD, heart attack, CHF, angina, CHD, and stroke was 12.3% (*n* = 735), 4.9% (*n* = 294), 3.7% (*n* = 222), 3.1% (*n* = 184), 4.9% (*n* = 293), and 4.0% (*n* = 238), respectively. The demographic characteristics in terms of CVD status are observed in Table 1. The CVD group presented a significantly higher age, more men participants, a higher proportion of non-Hispanic White individuals, lower levels of education and income, lower frequency of exercise, higher likelihood of smoking and drinking, and higher prevalence of obesity, hypertension, dyslipidemia, and diabetes.

**Table 1:** Baseline characteristics of study variables by CVD.

**Variables**	**CVD** **(*N* = 735)**	**Non-CVD** **(*N* = 5237)**	***P*-value**	**Total** **(*N* = 5972)**
Age, years	66.7 (12.3)	52.6 (14.4)	<0.001	54.2 (14.9)
Female, %	312 (42.4)	2784 (53.2)	<0.001	3096 (51.8)
Race/Ethnicity, %			<0.001	
Non-Hispanic White	413 (56.2)	2303 (44.0)		2716 (45.5)
Non-Hispanic Black	143 (19.5)	1078 (20.6)		1221 (20.4)
Mexican American	72 (9.8)	764 (14.6)		836 (14.0)
Other race	107 (14.6)	1092 (20.9)		1199 (20.1)
Education level, %			<0.001	
Less than 9th grade	123 (16.7)	590 (11.3)		713 (11.9)
9–11th grade	138 (18.8)	756 (14.4)		894 (15.0)
High school Grad/GED or equivalent	183 (24.9)	1139 (21.7)		1322 (22.1)
Some college or AA degree	175 (23.8)	1435 (27.4)		1610 (27.0)
College graduate or above	114 (15.5)	1309 (25.0)		1423 (23.8)
Missing	2 (0.3)	8 (0.2)		10 (0.2)
Annual household income, %			<0.001	
Under $20,000	235 (32.0)	1023 (19.5)		1258 (21.1)
$20,000 to $45,000	252 (34.3)	1701 (32.5)		1953 (32.7)
$45,000 to $75,000	122 (16.6)	915 (17.5)		1037 (17.4)
$75,000 to $100,000	47 (6.4)	481 (9.2)		528 (8.8)
Over $100,000	46 (6.3)	875 (16.7)		921 (15.4)
Missing	33 (4.5)	242 (4.6)		275 (4.6)
BMI, %			<0.001	
Underweight	10 (1.4)	65 (1.2)		75 (1.3)
Normal weight	139 (18.9)	1339 (25.6)		1478 (24.7)
Overweight	238 (32.4)	1829 (34.9)		2067 (34.6)
Obesity	332 (45.2)	1963 (37.5)		2295 (38.4)
Missing	16 (2.2)	41 (0.8)		57 (1.0)
Exercise regularly, %	76 (10.3)	892 (17.0)	<0.001	968 (16.2)
Current smoking, %	177 (24.1)	1105 (21.1)	0.012	1282 (21.5)
Current drinking, %	482 (65.6)	3373 (64.4)	0.026	3855 (64.6)
Hypertension, %	554 (75.4)	1931 (36.9)	<0.001	2485 (41.6)
Dyslipidemia, %	475 (64.6)	1785 (34.1)	<0.001	2260 (37.8)
Diabetes, %	222 (30.2)	616 (11.8)	<0.001	838 (14.0)

CVD, cardiovascular disease; BMI, body mass index; Continuous variables are presented as mean and SD. Categorical variables are presented as numbers and percentage.

Table S1 shows the demographic characteristics in terms of DCBA in quartiles. Compared with those in quartiles 1–3, participants in quartile 4 were more likely to be younger, men, non-Hispanic White, smoker, drinker, higher education levels, lower frequency of exercise, and lower prevalence of dyslipidemia, while the prevalence of hypertension and diabetes was not significantly different across quartiles 1–4. In addition, means of cardiovascular risk factors according to urinary DCBA concentrations are shown in Table 2. There were significant differences between DCBA quartiles in high-density lipoprotein cholesterol (HDL-C) and systolic blood pressure (SBP).

**Table 2:** Cardiovascular risk factors in terms of quartiles of differences in DCBA, NHANES 2007–2014.

	**DCBA (ug/L)**				***P-value***
	**Q1**	**Q2**	**Q3**	**Q4**	
FPG, mmol/L (*n =* 2808)	6.18 (2.09)	6.20 (2.15)	6.12 (2.04)	6.14 (2.23)	0.55
HbA1c, % (*n* = 5728)	5.87 (1.08)	5.88 (1.20)	5.83 (1.08)	5.83 (1.07)	0.13
TC, mmol/L (*n =* 5661)	5.17 (1.11)	5.10 (1.09)	5.11 (1.13)	5.14 (1.08)	0.51
HDL-C, mmol/L (*n =* 5661)	1.39 (0.41)	1.37 (0.43)	1.37 (0.43)	1.33 (0.41)	<0.001
LDL-C, mmol/L (*n =* 2717)	3.04 (0.91)	3.01 (0.93)	3.04 (0.91)	3.11(0.94)	0.17
SBP, mmHg (*n =* 5749)	126.9 (19.5)	125.2 (18.9)	125.5 (18.8)	124.0 (18.0)	<0.001
DBP, mmHg (*n =* 5749)	71.0 (13.0)	70.8 (12.7)	71.1 (13.4)	70.8 (13.0)	0.85

DCBA, 3-(diethlycarbamoyl) benzoic acid; FPG, fasting plasma glucose; HbA1c, glycohemoglobin; TC, total cholesterol; HDL-C, high-density lipoprotein cholesterol; LDL-C, low-density lipoprotein cholesterol; SBP, systolic blood pressure; DBP, diastolic blood pressure.

### Measurement of urinary DEET metabolites

Table 3 presents the detectable concentrations of DEET and its metabolites. DCBA was detected in 79.8% of participants, while detectable levels of DEET and DHMB were only 3.5 and 11.3%, respectively. In addition, the geometric mean concentration of DCBA was 2.58 μg/L.

**Table 3:** Distribution of urinary DEET and its metabolites (*N* = 5,972), NHANES 2007–2014.

**DEET metabolites (ug/L)**	**Detection frequency, *n* (%)**	**GM**	**5th**	**25th**	**50th**	**75th**	**95th**
DEET	211 (3.5 %)	0.06	< LOD	< LOD	< LOD	< LOD	< LOD
DHMB	673 (11.3 %)	0.08	< LOD	< LOD	< LOD	< LOD	0.67
DCBA	4766 (79.8 %)	2.58	< LOD	0.66	1.84	6.57	66.31

Detection frequency: the detection percentage of the population; LOD, limit of detection; GM, geometric mean; DEET, N,N-diethy-m-toluamide; DHMB, N,N-diethyl-3-(hydroxymethyl) benzamide; DCBA, 3-(diethlycarbamoyl) benzoic acid.

### Associations of DCBA with the risk of specific and total CVD

**Figure 1:**
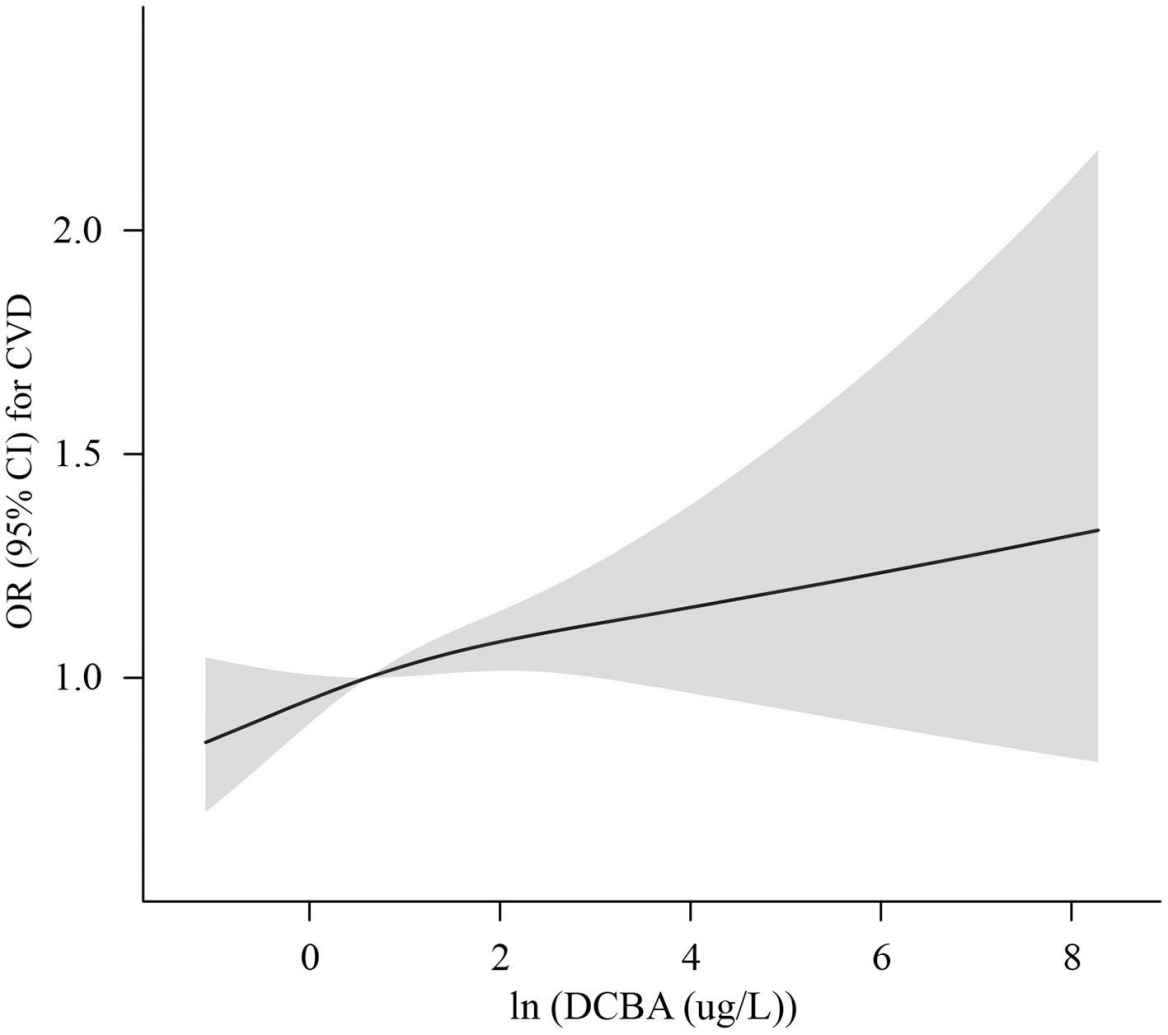
Adjusted OR (solid lines) with 95% CI (shaded areas) for CVD in urinary concentrations of (In-transformed) DCBA, Adjustments included age, sex, ethnicity, income, education, exercise, smoking, drinking, BMI, hypertension, dyslipidemia, diabetes, and In-transformed creatinine.

The association of DCBA exposure with total and specific CVD is manifested in Table 4. After adjustment for age, sex, ethnicity, education, income, smoking, drinking, exercise, BMI, hypertension, dyslipidemia, diabetes, and ln-transformed creatinine, high levels of DCBA exposure were associated with increased risks of CVD and CHD in adults. Compared to the lowest quartile, DCBA in the highest quartile was associated with an increased risk of CHD (OR 1.57; 95% CI: 1.10–2.25, *P* for trend = 0.017) and CVD (OR 1.32; 95% CI: 1.03–1.68, *P* for trend = 0.025). Except for CVD and CHD, no other specific CVD outcomes showed a positive association with DCBA in adults. In addition, RCS analysis flexibly modeled the relation of urinary DCBA with CVD. A positive correlation was manifested between urinary concentrations of DCBA and adjusted OR for CVD, with no evidence of a non-linear association between DCBA and OR for CVD, as shown in Figure 1 (*P* non-linearity = 0.49).

**Table 4:** Associations of urinary DCBA with total and specific CVD in adults.

**CVD events**	**DCBA (ug/L)**				***P–trend***
	**Q1**	**Q2**	**Q3**	**Q4**	
Heart attack (case/total)	71/1570	72/1418	70/1493	81/1491	
Model 1	1.00	1.21 (0.85–1.72)	1.18 (0.82–1.69)	1.40 (0.98–2.00)	0.12
Model 2	1.00	1.21 (0.85–1.72)	1.17 (0.82–1.68)	1.39 (0.97–1.98)	0.13
Model 3	1.00	1.22 (0.86–1.74)	1.19 (0.83–1.71)	1.44 (1.01–2.05)	0.088
CHF (case/total)	59/1570	57/1418	56/1493	50/1491	
Model 1	1.00	1.19 (0.81–1.75)	1.21 (0.82–1.80)	1.12 (0.74–1.69)	0.98
Model 2	1.00	1.16 (0.79–1.71)	1.17 (0.79–1.74)	1.10 (0.73–1.66)	0.99
Model 3	1.00	1.16 (0.78–1.71)	1.17 (0.78–1.75)	1.14 (0.75–1.73)	0.82
Angina (case/total)	52/1570	49/1418	37/1493	46/1491	
Model 1	1.00	1.09 (0.72–1.65)	0.82 (0.52–1.28)	1.05 (0.68–1.62)	0.76
Model 2	1.00	1.11 (0.74–1.68)	0.82 (0.52–1.28)	1.05 (0.68–1.62)	0.80
Model 3	1.00	1.12 (0.74–1.69)	0.83 (0.53–1.31)	1.09 (0.71–1.69)	0.65
CHD (case/total)	73/1570	71/1418	66/1493	83/1491	
Model 1	1.00	1.21 (0.85–1.72)	1.14 (0.79–1.64)	1.51 (1.06–2.15)	0.030
Model 2	1.00	1.21 (0.85–1.73)	1.12 (0.78–1.62)	1.50 (1.05–2.13)	0.035
Model 3	1.00	1.23 (0.86–1.76)	1.13 (0.79–1.64)	1.57 (1.10–2.25)	0.017
Stroke (case/total)	57/1570	52/1418	68/1493	61/1491	
Model 1	1.00	1.04 (0.70–1.55)	1.41 (0.96–2.06)	1.29 (0.87–1.91)	0.34
Model 2	1.00	0.99 (0.67–1.48)	1.36 (0.93–2.00)	1.26 (0.85–1.88)	0.33
Model 3	1.00	0.97 (0.65–1.45)	1.36 (0.92–2.01)	1.25 (0.84–1.87)	0.33
CVD (case/total)	190/1570	170/1418	181/1493	194/1491	
Model 1	1.00	1.05 (0.83–1.33)	1.15 (0.90–1.46)	1.29 (1.01–1.64)	0.041
Model 2	1.00	1.03 (0.81–1.31)	1.13 (0.89–1.44)	1.28 (1.00–1.63)	0.040
Model 3	1.00	1.05 (0.83–1.34)	1.15 (0.90–1.46)	1.32 (1.03–1.68)	0.025

DCBA, 3–(diethlycarbamoyl) benzoic acid; CHF, congestive heart failure; CHD, coronary heart disease; CVD, cardiovascular disease; Model 1 was adjusted for age, sex, ln–transformed creatinine; Model 2 was adjusted for Model 1 plus ethnicity, education, income, smoking, drinking, exercise, BMI; Model 3 was adjusted for Model 2 plus hypertension, dyslipidemia, and diabetes.

### Stratified and sensitivity analyses

Stratified associations of urinary DCBA with CVD by potential confounders are shown in Table S2. When CVD models were stratified by age (≤60 or >60 years), sex (male or female), race (non-Hispanic White or other race), income (low or high), and BMI (<30 or ≥30 kg/m^2^), no interaction tests were significant for DCBA after accounting for multiple testing. In sensitivity analyses, Table S3 presents the family history of CVD (heart attack) and diabetes to the models failed to influence the relationship between DCBA and total and specific CVD. In addition, there was no significant association between DCBA and CVD outcomes in weighted models, as shown in Table S4.

## Discussion

To the best of our knowledge, this is the first study to examine the association of exposure to DEET with the risk of CVD. In this general population-based study of the US adults, we found that urinary levels of DCBA were positively associated with increased risks of CVD and CHD. Our findings appeared to be supported by the following studies.

The potential effect of DEET on cardiovascular changes was found to be mainly concentrated in earlier human studies and animal experiments. One longitudinal health study of Gulf War veterans evaluated the health conditions of deployed veterans in the period of 1995–2005. Deployed Gulf War veterans had a significantly higher incidence risk ratio of CHD (1.61, 95% CI: 1.17–2.23) than non-deployed veterans by 2005, and the cardiovascular disorders have continuously worsened over a period of 10 years (20). It is reported that five patients exposed to concentrated DEET (47.5–95%) all had a symptom of hypotension that occurred within 1 h after ingestion, and two of them even died (20). In animal models, Leach et al. pointed out that intraperitoneal injections of DEET led to a marked decrease in blood pressure and heart rate (21). Research on canines showed the reduction of cardiac output, with no prominent change in stroke volume and peripheral resistance, which suggested that DEET-induced bradycardia was possibly the reason for hypotension (22). According to the pharmacological studies in rats, there was a significant decrease in the hypotension and heart rate from acetylcholine-following treatment with DEET. Hitherto, a lack of related evidence was provided to justify the DEET-induced hypotension but the cholinergic system could be suspected (10, 23).

Despite theprecise mechanisms that are responsible for the DEET-induced cardiovascular function are not fully characterized, the existing plausible interpretations can be entertained. DEET plays a pivotal role in the proliferation, migration, and adhesion of endothelial cells, and these effects were related to the expression of focal adhesion kinase phosphorylation, vascular endothelial growth factor, and nitric oxide production (24, 25), three main mediators regulated in angiogenesis. Nevertheless, the different specific molecular targets of DEET explaining its activity are still under debate. Acetylcholinesterase (AChE) activity was effectively inhibited by DEET and involved in the regulation of endothelial angiogenesis, as has been confirmed by an ischemic hindlimb model (26). Furthermore, the M3 muscarinic receptor was not only expressed in the nervous system, but it is known that this cholinergic receptor is also expressed in non-innervated tissues such as endothelial cells (27, 28). Importantly, the ability of DEET to increase angiogenic processes was susceptible to a muscarinic M3 receptor blockade or a decreased cellular expression of muscarinic M3 receptor, decreasing the release of carbachol-induced Ca^2+^ signal in endothelial cells (29). Recent evidence from *in vitro* and *in vivo* has revealed that DEET might increase acetylcholine bioavailability and bind to its M3 receptor through inhibiting AChE, thereby conducting proangiogenic effects by an allosteric modulation. In addition, in the perspective of epigenetics, the detrimental health effects of DEET are not limited to the individuals directly exposed but persist in multiple generations. In other words, future generations in the absence of exposure were very likely to increase disease incidence *via* epigenetic alterations through the germline of parents, such as DNA methylation (DNAm). Several rodent studies supported this point and demonstrated significant DNAm changes of transgenerational disease present in sperm (30, 31). Unfortunately, since the inheritable phenotypes that were related to the cardiovascular system were rarely reported, the potential mechanism of DEET on CVD needs to be cautiously elucidated.

The strength of this study is first to examine the association of DEET exposure with specific and total CVD risk using high-precise urinary sample data from a well-designed population-based study (NHANES). Nevertheless, there were several limitations in this study. First, the nature of this study is cross-sectional and this study indicates the fact that causal association between DEET exposure and CVD could not be established. Hence, prospective cohort studies are required to provide more evidence. Second, although reporters regarding the half-lives of DEET in humans were sparse, it is documented that the half-lives of DEET in most mammals were within 6 h for the reference, meaning that DEET in urine is rapidly metabolized and eliminated from the body (32–35). Thus, it is unclear whether a single measurement may reflect long-term exposure. Third, the outcome variables based on self-reported outcome variables might contribute to the misclassification of some individuals. Fourth, we try to control a series of the potential risk factors in the statistical analyses, but the relationship of DEET exposure with CVD prevalence in adults ought to be assessed with caution, because, in addition to DEET, some other confounders, such as genetic factors, may also have influenced CVD prevalence and need to be considered in future studies. Fifth, urinary concentrations of DCBA were detectable in most persons, while DEET and DHMB were, respectively, detected only in 3.5 and 11.3% of them in this study. As a result, we evaluated the association between CVD and DCBA rather than DEET. Finally, the study did not use the NHANES sampling parameters; hence, our findings cannot be generalized beyond this group.

In conclusion, this study found that exposure to DEET may contribute to increased risks of CHD and CVD in adults, which provide strong evidence of DEET-induced cardiovascular changes in humans. Although two prior large-scale analyses based on the US Poison Center data deduced that DEET had less risk when used in accordance with the product labels (36, 37), considering that individual differences in the absorption ability of DEET exist, and it is necessary to inform the people about these risks and to raise the public awareness.

## Data availability statement

Publicly available datasets were analyzed in this study. This data can be found here: https://wwwn.cdc.gov/nchs/nhanes/Default.aspx.

## Ethics statement

The studies involving human participants were reviewed and approved by National Center for Health Statistics. The patients/participants provided their written informed consent to participate in this study.

## Author contributions

WW and SH conceived and designed the idea for the study. YW and ZiW arranged a series of procedures to achieve population data. SY analyzed the data, interpreted the results, and wrote the manuscript. All authors were responsible for revising the manuscript and approving the final version.

## Funding

This research was supported by the HMU Marshal Initiative Funding (HMUMIF-21011 to WJ) and the National Natural Science Foundation of China (81872615 to SH).

## Conflict of interest

The authors declare that the research was conducted in the absence of any commercial or financial relationships that could be construed as a potential conflict of interest.

## Publisher's note

All claims expressed in this article are solely those of the authors and do not necessarily represent those of their affiliated organizations, or those of the publisher, the editors and the reviewers. Any product that may be evaluated in this article, or claim that may be made by its manufacturer, is not guaranteed or endorsed by the publisher.
