# Supplementary material for: Exposure to N,N-diethyl-m-toluamide and cardiovascular diseases in adults
Source: Front Public Health. 2022 Oct 3;10:922005. doi: 10.3389/fpubh.2022.922005 (PMC9576625; doi:10.3389/fpubh.2022.922005)
Supplement: Supplementary file 1 [file Table_1.docx]

| Characteristics | DCBA (ug/L) | | | | *P-value* |
| --- | --- | --- | --- | --- | --- |
|  | Q1 | Q2 | Q3 | Q4 |  |
| Number of participants | 1570 | 1418 | 1493 | 1491 |  |
| Age, years | 56.4 (14.7) | 54.8 (14.9) | 53.4 (14.9) | 52.7 (14.8) | <0.001 |
| Female, % | 888 (56.6) | 767 (54.1) | 754 (50.5) | 687 (46.1) | <0.001 |
| Race/Ethnicity, % |  |  |  |  | <0.001 |
| Non-Hispanic White | 702 (44.7) | 637 (44.9) | 643 (43.1) | 734 (49.2) |  |
| Non-Hispanic Black | 214 (13.6) | 315 (22.2) | 362 (24.2) | 330 (22.1) |  |
| Mexican American | 247 (15.7) | 183 (12.9) | 202 (13.5) | 204 (13.7) |  |
| Other | 407 (25.9) | 283 (20.0) | 286 (19.2) | 223 (15.0) |  |
| Education level, % |  |  |  |  | <0.01 |
| Less than 9th grade | 215 (13.7) | 175 (12.3) | 167 (11.2) | 156 (10.5) |  |
| 9-11th grade | 201 (12.8) | 196 (13.8) | 243 (16.3) | 254 (17.0) |  |
| High school Grad/GED or equivalent | 324 (20.6) | 322 (22.7) | 325 (21.8) | 351 (23.5) |  |
| Some college or AA degree | 405 (25.8) | 388 (27.4) | 433 (29.0) | 384 (25.8) |  |
| College graduate or above | 421 (26.8) | 335 (23.6) | 322 (21.6) | 345 (23.1) |  |
| Missing | 4 (0.3) | 2 (0.1) | 3 (0.2) | 1 (0.1) |  |
| Annual household income, % |  |  |  |  | 0.019 |
| Under $20,000 | 300 (19.1) | 305 (21.5) | 359 (24.0) | 294 (19.7) |  |
| $20,000 to $45,000 | 494 (31.5) | 459 (32.4) | 476 (31.9) | 524 (35.1) |  |
| $45,000 to $75,000 | 291 (18.5) | 241 (17.0) | 265 (17.7) | 240 (16.1) |  |
| $75,000 to $100,000 | 140 (8.9) | 125 (8.8) | 135 (9.0) | 128 (8.6) |  |
| Over $100,000 | 265 (16.9) | 218 (15.4) | 194 (13.0) | 244 (16.4) |  |
| Missing | 80 (5.1) | 70 (4.9) | 64 (4.3) | 61 (4.1) |  |
| BMI, % |  |  |  |  | <0.001 |
| Underweight | 21 (1.3) | 16 (1.1) | 18 (1.2) | 20 (1.3) |  |
| Normal weight | 459 (29.2) | 350 (24.7) | 338 (22.6) | 331 (22.2) |  |
| Overweight | 549 (35.0) | 492 (34.7) | 521 (34.9) | 505 (33.9) |  |
| Obesity | 523 (33.3) | 546 (38.5) | 601 (40.3) | 625 (41.9) |  |
| Missing | 18 (1.1) | 14 (1.0) | 15 (1.0) | 10 (0.7) |  |
| Regular exercise, % | 305 (19.4) | 214 (15.1) | 239 (16.0) | 210 (14.1) | <0.001 |
| Current smoking, % | 265 (16.9) | 270 (19.0) | 358 (24.0) | 389 (26.1) | <0.001 |
| Current drinking, % | 962 (61.3) | 895 (63.1) | 961 (64.4) | 1037 (69.6) | <0.001 |
| Hypertension, % | 648 (41.3) | 594 (41.9) | 632 (42.3) | 611 (41.0) | 0.21 |
| Dyslipidemia, % | 625 (39.8) | 538 (37.9) | 556 (37.2) | 541 (36.3) | <0.001 |
| Diabetes, % | 222 (14.1) | 207 (14.6) | 211 (14.1) | 198 (13.3) | 0.47 |

**Table S1.** Baseline characteristics in terms of quartiles of differences in DCBA: NHANES, 2007–2014.

DCBA, 3-(diethlycarbamoyl) benzoic acid; CVD, cardiovascular disease; BMI, body mass index; Continuous variables are presented as mean and SD. Categorical variables are presented as numbers and percentage.
