# Supplementary material for: Exposure to N,N-diethyl-m-toluamide and cardiovascular diseases in adults
Source: Front Public Health. 2022 Oct 3;10:922005. doi: 10.3389/fpubh.2022.922005 (PMC9576625; doi:10.3389/fpubh.2022.922005)
Supplement: Supplementary file 2 [file Table_2.pdf]

Table S2. Stratified analyse of the associations between urinary concentrations of DCBA and CVD in NHANES 2007-2014.

	DCBA (ug/L)				*P*-*interaction*
	Q1	Q2	Q3	Q4	
Age					0.86
<60	1.00	1.14 (0.70-1.85)	1.11 (0.68-1.79)	1.29 (0.80-2.10)	
≥60	1.00	1.06 (0.80-1.39)	1.15 (0.87-1.53)	1.35 (1.02-1.80)	
Sex					0.90
Male	1.00	1.28 (0.91-1.80)	1.10 (0.78-1.55)	1.43 (1.02-1.99)	
Female	1.00	0.82 (0.58-1.18)	1.18 (0.83-1.68)	1.12 (0.77-1.63)	
Race					0.25
Non-Hispanic White	1.00	0.83 (0.59-1.16)	0.91 (0.64-1.27)	1.12 (0.81-1.56)	
Other race	1.00	1.38 (0.96-1.97)	1.50 (1.05-2.15)	1.49 (1.03-2.17)	
Income					0.78
Low	1.00	1.02 (0.75-1.38)	1.16 (0.86-1.57)	1.47 (1.08-1.99)	
High	1.00	1.12 (0.73-1.73)	1.06 (0.67-1.68)	1.04 (0.66-1.65)	
BMI					0.061
<30	1.00	1.12 (0.81-1.56)	1.22 (0.88-1.69)	1.37 (1.00-1.92)	
≥30	1.00	0.90 (0.62-1.32)	1.02 (0.70-1.48)	1.20 (0.82-1.74)	

DCBA, 3-(diethlycarbamoyl) benzoic acid; CVD, cardiovascular disease; BMI, body mass index; Income (low: <$75000, high: >$75000) Adjustments included age, sex, ethnicity, education, income, smoking, drinking, exercise, BMI, hypertension, dyslipidemia, diabetes, and ln-transformed creatinine. The strata variable was not included in the model when stratifying by itself.
