# Supplementary material for: Exposure to N,N-diethyl-m-toluamide and cardiovascular diseases in adults
Source: Front Public Health. 2022 Oct 3;10:922005. doi: 10.3389/fpubh.2022.922005 (PMC9576625; doi:10.3389/fpubh.2022.922005)
Supplement: Supplementary file 3 [file Table_3.pdf]

Table S3. Sensitivity Analyse: Association of urinary DCBA with total and specific CVD in adults after additional adjustment for family history of CVD and diabetes .

CVD events	DCBA (ug/L)				*P-trend*
	Q1	Q2	Q3	Q4	
Heart attack	1.00	1.21 (0.85-1.72)	1.19 (0.83-1.71)	1.43 (1.00-2.04)	0.091
CHF	1.00	1.15 (0.78-1.71)	1.17 (0.78-1.75)	1.13 (0.75-1.72)	0.83
Angina	1.00	1.11 (0.74-1.69)	0.83 (0.53-1.30)	1.09 (0.70-1.68)	0.67
CHD	1.00	1.23 (0.86-1.76)	1.13 (0.78-1.64)	**1.57 (1.10-2.25)**	0.017
Stroke	1.00	0.96 (0.64-1.44)	1.37 (0.93-2.01)	1.25 (0.83-1.86)	0.34
CVD	1.00	1.05 (0.82-1.33)	1.15 (0.90-1.46)	**1.31 (1.03-1.68)**	0.025

DCBA, 3-(diethlycarbamoyl) benzoic acid; CHF, congestive heart failure; CHD, coronary heart disease; CVD, cardiovascular disease; Adjustments included age, sex, ln-transformed creatinine, ethnicity, education, income, smoking, drinking, exercise, BMI, hypertension, dyslipidemia, diabetes, and family history of CVD (heart attack) and diabetes.
