# Supplementary material for: Exposure to N,N-diethyl-m-toluamide and cardiovascular diseases in adults
Source: Front Public Health. 2022 Oct 3;10:922005. doi: 10.3389/fpubh.2022.922005 (PMC9576625; doi:10.3389/fpubh.2022.922005)
Supplement: Supplementary file 4 [file Table_4.pdf]

Table S4. Sensitivity Analyse: Associations of urinary DCBA with total and specific CVD in adults (weighted).

CVD events	DCBA (ug/L)			
	Q1	Q2	Q3	Q4
Heart attack (case/n)	71/1570	72/1418	70/1493	81/1491
Model 1	1.00	0.82 (0.54-1.24)	0.82 (0.52-1.29)	0.79 (0.51-1.21)
Model 2	1.00	0.86 (0.57-1.29)	0.84 (0.54-1.32)	0.79 (0.52-1.21)
Model 3	1.00	0.83 (0.55-1.25)	0.83 (0.53-1.30)	0.78 (0.52-1.19)
CHF (case/n)	59/1570	57/1418	56/1493	50/1491
Model 1	1.00	0.94 (0.49-1.82)	0.82 (0.45-1.50)	0.85 (0.48-1.50)
Model 2	1.00	0.93 (0.49-1.77)	0.82 (0.46-1.48)	0.84 (0.48-1.48)
Model 3	1.00	0.90 (0.46-1.71)	0.80 (0.44-1.47)	0.84 (0.48-1.48)
Angina (case/n)	52/1570	49/1418	37/1493	46/1491
Model 1	1.00	1.05 (0.59-1.85)	1.22 (0.68-2.18)	0.85 (0.47-1.51)
Model 2	1.00	1.06 (0.61-1.86)	1.23 (0.68-2.20)	0.83 (0.47-1.48)
Model 3	1.00	1.01 (0.58-1.78)	1.19 (0.67-2.11)	0.83 (0.46-1.49)
CHD (case/n)	73/1570	71/1418	66/1493	83/1491
Model 1	1.00	0.70 (0.48-1.01)	0.97 (0.65-1.45)	0.84 (0.60-1.18)
Model 2	1.00	0.70 (0.49-1.01)	0.97 (0.65-1.46)	0.83 (0.59-1.17)
Model 3	1.00	0.67 (0.46-0.97)	0.97 (0.65-1.45)	0.84 (0.60-1.18)
Stroke (case/n)	57/1570	52/1418	68/1493	61/1491
Model 1	1.00	1.24 (0.81-1.91)	0.88 (0.55-1.41)	1.15 (0.71-1.87)
Model 2	1.00	1.27 (0.81-2.01)	0.90 (0.57-1.42)	1.19 (0.74-1.89)
Model 3	1.00	1.26 (0.81-1.98)	0.88 (0.56-1.40)	1.16 (0.71-1.91)
CVD (case/n)	190/1570	170/1418	181/1493	194/1491
Model 1	1.00	0.96 (0.73-1.27)	0.95 (0.70-1.29)	1.10 (0.86-1.40)
Model 2	1.00	0.96 (0.73-1.26)	0.95 (0.71-1.27)	1.09 (0.85-1.39)
Model 3	1.00	0.93 (0.71-1.23)	0.95 (0.71-1.27)	1.08 (0.85-1.38)

DCBA, 3-(diethlycarbamoyl) benzoic acid; CHF, congestive heart failure; CHD, coronary heart disease; CVD, cardiovascular disease; Model 1 was adjusted for age, sex, ln-transformed creatinine; Model 2 was adjusted for Model 1 plus ethnicity, education, income, smoking, drinking, exercise, BMI; Model 3 was adjusted for Model 2 plus hypertension, dyslipidemia, and diabetes.
