# Supplementary figures and images for: Exposure to N,N-diethyl-m-toluamide and cardiovascular diseases in adults
Source: Front Public Health. 2022 Oct 3;10:922005. doi: 10.3389/fpubh.2022.922005 (PMC9576625; doi:10.3389/fpubh.2022.922005)

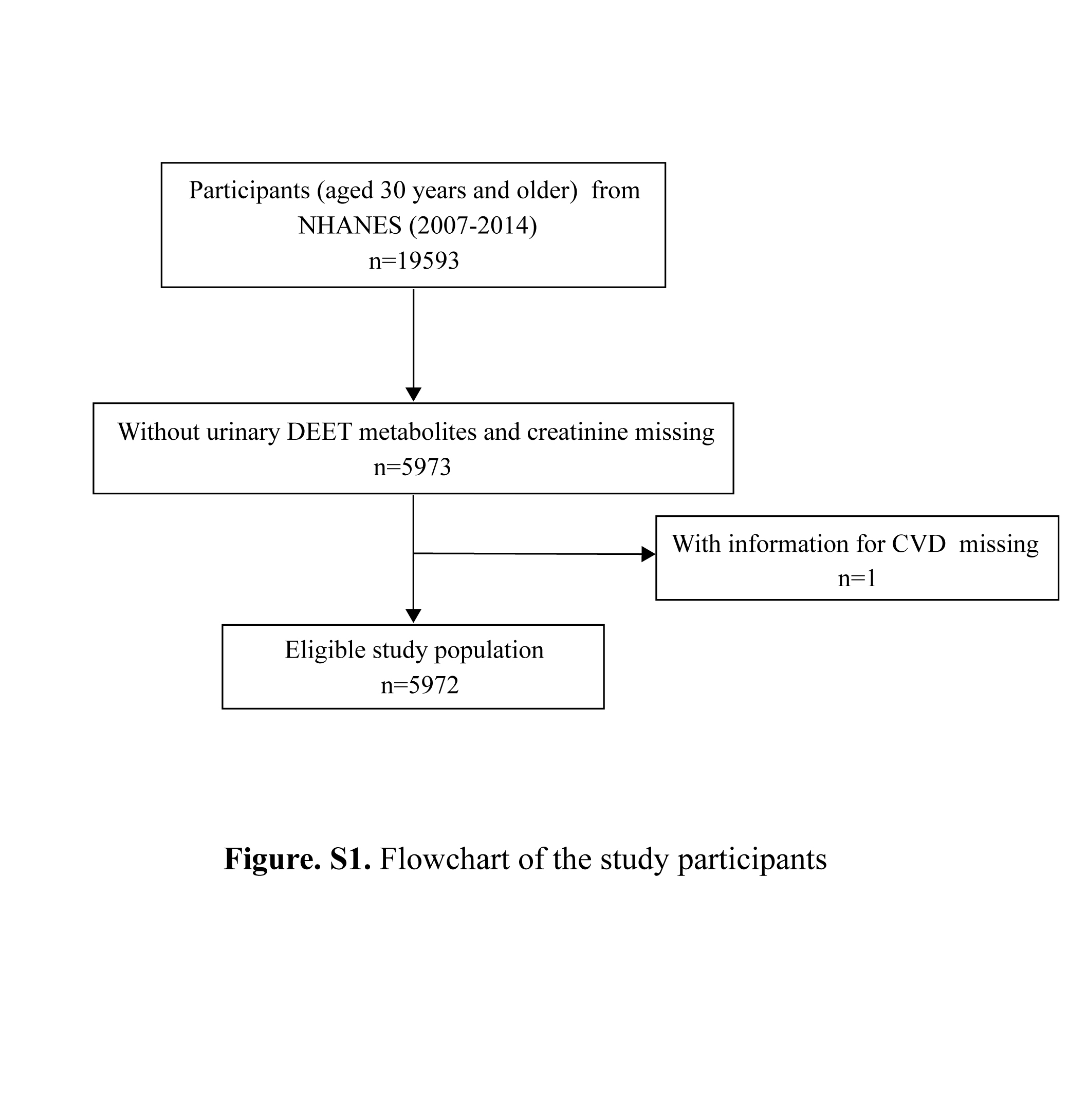

Supplement: Supplementary file 5 [file Image_1.JPEG]
